# Understanding and Addressing Delays in Endocrine Surgery: Insights From a Mixed‐Methods Study and a Pilot Educational Intervention

**DOI:** 10.1002/jso.70341

**Published:** 2026-07-16

**Authors:** Stella Kim, Maxwell Foote, Sophia Zhang, Audrey Twyford, Abigail Fox, Jennifer E. Rosen

**Affiliations:** ^1^ Georgetown University School of Medicine Washington District of Columbia USA; ^2^ Department of Endocrine Surgery MedStar Washington Hospital Center Washington District of Columbia USA; ^3^ Georgetown University Washington District of Columbia USA

**Keywords:** communication, delays in care, education, endocrine surgery, interoperability, referral

## Abstract

**Background and Objectives:**

Delays in endocrine surgical care contribute to adverse events, patient dissatisfaction, and mortality. Communication is an overlooked contributor. This study identified multi‐level drivers of delay and piloted a provider‐focused intervention.

**Methods:**

We conducted an IRB‐approved mixed‐methods study. A retrospective analysis of 898 patients who underwent thyroid, parathyroid, and adrenal surgery at a single academic center between January 2023 and August 2024 quantified referral (first abnormal lab or imaging to first surgical consultation) and treatment (consultation to surgery) delays, evaluating sociodemographic, patient portal, and interoperability factors. Semi‐structured interviews with patients and staff explored patient‐, provider‐, and system‐level barriers. A pilot educational intervention for primary care providers (PCPs) assessed pre‐post changes in referral‐related knowledge and comfort caring for primary hyperparathyroidism.

**Results:**

Median referral delay (157 days) exceeded treatment delay (62 days), suggesting delays occurred primarily during the referral phase of care. Older age, female sex, and thyroid disease independently predicted longer referral delays. Our educational intervention improved PCP recognition and referral confidence.

**Conclusions:**

Delays in endocrine surgical care are multifactorial, spanning patient‐, provider‐, and system‐level factors. Provider‐focused interventions are promising, but prospective studies are needed to confirm impact on delays and outcomes.

## 1 Introduction

Significant changes in endocrine surgical care delivery have occurred in recent years, including deferral of elective operations, reduction in practice volumes, and increased reliance on routine surveillance [1]. Some early studies suggested that short‐term delays in endocrine procedures may be acceptable. For example, Collins et al. reported that 4% of patients experienced disease progression and 4.1% required a change in the operative plan, with no significant association between delay duration and disease progression [2].

However, more recent and larger studies challenge this narrative that delays in thyroid cancer surgery are uniformly benign. In a 2023 analysis by Chaves et al., delayed surgery longer than 180 days was associated with worse disease‐specific survival (DSS) in patients with localized disease (aHR 3.51; 95% CI, 1.68–7.32), although no significant associations were observed in patients with regional or metastatic disease, and whole study population demonstrated no difference in DSS with increasing time to surgery [3]. Similar trends are observed in parathyroid disease, where surgical delay > 2 years increased the risk of renal stone treatment by 59%, coronary disease by 118%, and overall cardiovascular events by 52% [4].

Emerging evidence suggests that both patient‐level and systems‐level factors contribute to these delays. Older age and residence in socially vulnerable communities were associated with up to 300 days longer wait times to parathyroidectomy [5]. Race, insurance status, and distance from the treatment facility have been linked to delayed thyroid surgery and higher stage at presentation [6]. In adrenal surgery, nearly 43% of patients experienced delayed adrenalectomy, most commonly due to delayed hormonal workup [7].

Communication is an integral yet often overlooked contributor. Ineffective communication across patients, providers, and institutions can lead to adverse events, treatment delays, medical errors, wrong‐site surgery, decreased care continuity, patient dissatisfaction, and mortality [8]. With increasing reliance on electronic health records and virtual platforms, communication is central at interpersonal, institutional, and system‐wide levels [1]. Secure messaging, provider portals, and health information exchange improve access to critical information and integration, particularly when used in combination [9]. However, despite high patient satisfaction with telemedicine, delays persist, suggesting that technology alone is insufficient to mitigate delays in endocrine surgery [10].

Accordingly, we conducted a convergent mixed‐methods study to better understand how communication and multi‐level factors contribute to delays in endocrine surgical care at our institution. Additionally, we piloted a provider‐focused educational intervention aimed at improving referral processes, with the goal of reducing delays and optimizing patient outcomes.

## 2 Materials and Methods

### 2.1 Ethical Approval

This mixed‐methods study was conducted in three stages to evaluate multi‐level contributors to delays in endocrine surgical care and assess a pilot intervention. Ethical approval was granted by the Georgetown University‐MedStar Health Institutional Review Board (#00008179, #00008236, #00010121). All research practices were carried out in accordance with the Helsinki Declaration of 1975, as revised in 1983.

### 2.2 Retrospective Quantitative Analysis

The first stage included 898 patients who underwent surgery for thyroid, parathyroid, or adrenal diseases between January 2023 and August 2024. Patients were identified through a reverse search of the MedStar Health System database using CPT codes. Electronic medical records were reviewed to collect demographic information, including race, age, gender, marital status, portal enrollment, and participation in health information exchanges (CRISP or CommonWell). Portal enrollment was defined as an active account regardless of usage. CRISP, or the Chesapeake Regional Information System for our Patients, is a non‐profit health information exchange that securely shares clinical information among healthcare providers in Maryland and Washington, DC [11]. CommonWell Health Alliance is a nationwide health data exchange and interoperability network [12]. The date of diagnosis was defined as the first date of abnormal imaging or laboratory results. Delay in referral was calculated as the interval from diagnosis to first surgical consultation, and delay in treatment was defined as the interval from consultation to surgery.

Quantitative analysis was performed in STATA BE 19.5 (StataCorp, College Station, TX). Categorical variables were summarized with frequencies and percentages. Continuous variables were assessed for normality using Shapiro‐Wilk tests and reported as means and standard deviation (SD) or medians and interquartile range (IQR). Univariate analysis included chi‐square tests, Mann‐Whitney U, Kruskal‐Wallis, or Spearman correlation. Skewed variables were log‐transformed for multivariate regression with back‐transformed results reported for interpretability. Statistical significance was set at *p* < 0.05, and two‐tailed test results are reported.

### 2.3 Prospective Qualitative Interviews

Concurrently, 15 semi‐structured interviews were conducted with English‐speaking patients and staff over 18 years old. Patients were treated for thyroid (*n* = 5), parathyroid (*n* = 3), or adrenal (*n* = 3) disease. Staff participants included a medical assistant, front desk staff, ambulatory nurse coordinator, and administrative coordinator. Verbal informed consent was obtained prior to interviews, with participants given the option not to be recorded.

Interviews explored patient experiences, communication, referral processes, clinic workflow, staff roles, and perceptions of barriers and facilitators to timely care. Interviews were recorded, transcribed, stored securely, and analyzed thematically using an iterative coding process to identify patterns at the patient, provider, and system levels.

### 2.4 Pilot Intervention

Following analysis of the quantitative and qualitative data, a pilot educational intervention was implemented for primary care providers (PCPs). PCPs attended a 1‐hour grand rounds session led by an endocrine surgeon who presented a case scenario of a patient with primary hyperparathyroidism covering indications for surgery, referral pathways, communication strategies, and system resources. Participants received direct clinic contact information and referral guidelines afterwards.

Pre‐ and post‐intervention surveys collected PCP demographic information, such as age, gender identity, race, and years in practice. PCP self‐reported their comfort identifying patients who require a hypercalcemia evaluation on a 5‐point Likert scale. Participants also ranked their familiarity with potential complications of untreated hypercalcemia, comfort in communicating urgency to patients, and likelihood of referring a standard patient with serum calcium of 10.4 mg/dL and PTH of 65 pg/mL. Respondents were also asked to indicate perceived barriers to referral. Survey responses were summarized using descriptive statistics in STATA BE 19.5 (StataCorp, College Station, TX). Pre‐ and post‐intervention group averages were compared without matching in accordance with IRB guidelines.

## 3 Results

### 3.1 Patient Characteristics and Quantitative Findings

Among 898 patients, 79.3% (712/898) were female and 20.7% (186/898) were male, with a mean age of 55.6 years (SD = 15.3). Surgical indications included thyroid disease in 50.8% (456/898), parathyroid disease in 47.6% (427/898), and adrenal disease in 1.6% (14/898). Racial distribution was 46.0% White (413/898), 37.8% Black or African American (340/898), and 16.2% other or multiple races, Asian or Pacific Islander, or declined to answer (145/898). Marital status included 53.2% married (478/898) and 46.8% not married (420/898). Portal enrollment was 74.6% (670/898), and 81.7% (734/898) were enrolled in CRISP or CommonWell. These findings are summarized in Table 1.

**Table 1:** Descriptive statistics of patient population.

Variables	N = 898
**Gender**	
Female (*n* = 712)	79.3%
Male (*n* = 186)	20.7%
**Age** (Mean ±SD)	55.6 ± 15.3
**Surgery Type**	
Thyroid (*n* = 456)	50.8%
Parathyroid (*n* = 427)	47.6%
Adrenal (*n* = 14)	1.6%
**Race**	
White (*n* = 413)	46.0%
Black or African American (*n* = 340)	37.8%
Other (*n* = 145)	16.2%
**Marital Status**	
Married (*n* = 478)	53.2%
Single (*n* = 420)	46.8%
**Portal Use**	
Enrolled (*n* = 670)	74.6%
Not enrolled (*n* = 228)	25.4%
**CRISP or CommonWell Enrollment**	
Enrolled (*n* = 734)	81.7%
Not enrolled (164)	18.3%

Median delays in referral were 157 days (range: 10–6101). Median delays in treatment were 62 days (range: 8–2244). Univariate analysis shows that portal enrollment is significantly associated with shorter referral delays (median 142 days vs. 203 days, *p* = 0.02). Female patients experienced longer delays in referral (176 vs. 111.5 days, *p* < 0.0001) and delays in treatment (64.5 vs. 54 days, *p* = 0.01). Additionally, age was significantly associated with delays in referral (*p* < 0.0001) and treatment (*p* < 0.0001) as older patients experienced greater delays in referral, although the association was very weak (Spearman's rho = 0.14 and 0.13, respectively). Black or African American patients (median 72 days) experienced longer delays in treatment (*p* < 0.0001) compared to White (median 72 days vs. 57 days) and other races (58 days). These findings are summarized in Table 2.

**Table 2:** Univariate associations with delays in care.

	Delays in referral	*p*‐value	Delays in treatment	*p*‐value
**Gender** (Median, IQR)		< 0.0001*		0.01*
Female	176 [69, 578]		64.5 [40, 116]	
Male	111.5 [50, 281]		54 [36, 109]	
**Age** (Spearman's rho)	0.14	< 0.0001*	0.13	< 0.0001*
**Surgery Type** (Median, IQR)		0.50		0.21
Thyroid	161 [65.5, 601.5]		66.5 [37, 122]	
Parathyroid	149.5 [63, 432]	59 [40, 103]		
Adrenal	114 [49, 619]		96.5 [43, 227]	
**Race** (Median, IQR)		0.06		< 0.0001*
White	142 [57, 509]		57 [37,94]	
Black or African American	187 [71, 610]		72 [44, 161.5]	
Other	127 [66, 415]		58 [34, 109]	
**Marital Status**		0.52		0.07
(Median, IQR)Married	160.5 [65, 530]		60 [37, 105]	
Single	151 [61, 513]		65 [41, 126]	
**Portal Use** (Median, IQR)		0.02*		0.73
Portal Use	142 [81, 556]		62 [38, 112]	
No Portal Use	203 [57, 556]		61.5 [38,121.5]	
**CRISP or Commonwell Enrollment** (Median, IQR)		0.57		0.60
Enrollment	135.5 [63.5, 525]		62 [34.5, 108.5]	
No enrollment	161 [63, 525]		62 [39, 116]	

*p < 0.05 for statistically significant results.

Multivariate linear regression identified independent predictors of delays in referral and treatment, adjusting for gender, age, surgery type, race, marital status, portal enrollment, and CRISP or CommonWell enrollment. For delays in referral, female patients experienced 60% longer delays than male patients (*p* < 0.0001), and each additional year of age increased delay by 1.5% (*p* < 0.0001). Patients undergoing parathyroid surgery had 19% shorter delays in referral compared to those undergoing thyroid surgery (*p* = 0.045). Although portal enrollment was associated with shorter referral delays in univariate analysis, this association was no longer significant after adjustment for covariates (*p* > 0.05). Race, marital status, and CRISP or CommonWell enrollment were not significantly associated with referral delays in the multivariate analysis. These results are shown in Table 3. For delays in treatment, each additional year of age was associated with 1% longer treatment delay (*p* < 0.0001). Patients undergoing parathyroid surgery experienced 13% shorter delays in treatment compared to those undergoing thyroid surgery (*p* = 0.03). Black or African American patients experienced 27% longer delays in treatment compared to White patients (*p* < 0.0001) and 26% longer delays compared to patients of other races (*p* = 0.003). Portal enrollment, gender, marital status, and CRISP or CommonWell enrollment were not significantly associated with treatment delays in multivariate analysis (*p* > 0.05). These findings are summarized in Table 4.

**Table 3:** Multivariate analysis for delays in referral.

Variable	Coefficient (CI)	*p*‐value
**Gender**	0.47 (0.22, 0.71)	< 0.0001*
**Age**	0.015 (0.0080, 0.022)	< 0.0001*
**Surgery Type**		
Thyroid versus Parathyroid	−0.21 (‐0.41, ‐0.005)	0.045*
Thyroid versus Adrenal	−0.082 (‐0.88, 0.72)	0.84
Parathyroid versus Adrenal	−0.13 (‐0.93, 0.68)	0.76
**Race**		
African American versus White	−0.19 (‐0.41, 0.041)	0.11
African American versus Other	−0.06 (‐0.37, 0.24)	0.68
White versus Other	−0.12 (‐0.41, 0.17)	0.42
**Marital Status**	0.10 (‐0.10, 0.31)	0.32
**Portal Use**	−0.45 (‐0.28, 0.19)	0.71
**CRISP or Commonwell Enrollment**	0.085 (0.008, 0.022)	0.52

*p < 0.05 for statistically significant results.

**Table 4:** Multivariate analysis for delays in treatment.

Variable	Coefficient (CI)	p‐value
**Gender**	0.16 (−0.00011, 0.32)	0.05
**Age**	0.010 (0.0055, 0.014)	< 0.0001*
**Surgery Type**		
Thyroid versus Parathyroid	−0.14 (−0.27, −0.010)	0.03*
Thyroid versus Adrenal	0.22 (−0.29, 0.73)	0.40
Parathyroid versus Adrenal	−0.36 (−0.88, 0.15)	0.17
**Race**		
African American versus White	−0.31 (−0.45, −0.16)	< 0.0001*
African American versus Other	−0.30 (−0.49, −0.10)	0.003*
White versus Other	−0.011 (−0.20, 0.18)	0.91
**Marital Status**	−0.10 (−0.24, 0.027)	0.12
**Portal Use**	0.11 (−0.42, 0.26)	0.16
**CRISP or Commonwell Enrollment**	−0.005 (−0.12, 00.26)	0.95

*p < 0.05 for statistically significant results.

### 3.2 Prospective Qualitative Analysis

Thematic analysis identified multi‐level factors across patient, provider, and system domains. Patient‐level barriers included holidays, anxiety, and competing life priorities. At the clinical level, diagnostic uncertainty and periods of watchful waiting contributed to prolonged timelines, particularly in patients with primary hyperparathyroidism whose hypercalcemia was missed or attributed to a different cause for years. System‐level constraints posed the greatest barrier, as limited clinic availability and challenges navigating complex care pathways involving multiple providers, appointments, and tests created problems in access to specialty care, contributing to delays and patient dissatisfaction. These qualitative findings aligned with quantitative results demonstrating longer delays in referral prior to surgical consultation, followed by more streamlined progression to surgery once specialty care was established.

Participants described several methods of communication across the care continuum. Staff‐patient communication occurred through phone calls, in‐person discussions, patient portals, printed materials, and surveys. Communication between providers relied on in‐person interactions, electronic medical record messaging, email, phone calls, fax, health information exchanges (CRISP/CommonWell), and at times patients serving as intermediaries. Despite the availability of multiple communication modalities, many staff noted that keeping track of various modes of communication was a demanding aspect of their job responsibilities.

Participants highlighted several themes regarding the strengths and weaknesses of their communication experiences. Strengths included active listening resulting in proactiveness, clear and patient‐centered communication, as well as professionalism and respect. First, active listening and proactive outreach helped patients feel cared for and at ease, with elements such as genuine interest in concerns, initial rapport, small talk and jokes, or targeted questions enhancing trust. One patient recounted, “I remember getting more than one email and a phone call…they did kind of reach out to me until they heard from me.” Second, patient‐tailored explanations of complex ideas using simple language and analogies were also valued by multiple patients emphasizing the importance of meeting them at their knowledge level. One patient expressed that a competent physician who clearly explains their diagnosis was more important than one who is simply “warm and fuzzy.” Third, professionalism and respect did not go unnoticed by the patients despite the busy and complex environment of a medical setting. One patient remarked that “staff can make or break a doctor's office, because if they're kind of haphazard and they behave as though you don't really matter, somebody like me isn't coming back.”

Weaknesses included lack of anticipatory guidance, miscommunication, and lack of accessibility. First, lack of anticipatory guidance was noted by one patient, who felt that having a family member in healthcare allowed them to understand what to expect next. They concluded that their situation would have been very difficult to navigate without their level of health literacy and trust in the healthcare system. Second, miscommunication undermines patients’ confidence in the system. Correct patient identification and follow‐through of promised post‐operative follow‐up were crucial not only for patient safety but for satisfaction as well. One staff member described how limited communication between central scheduling and the clinic front desk sometimes led to incorrect appointments, resulting in cancellations and workflow inefficiencies. Third, accessibility was noted as a barrier to communication. Patients valued direct person‐to‐person contact with the office, as opposed to automated or centralized phone systems. Telehealth was highlighted as a potential solution, offering timely and convenient access for questions, follow‐up, and care coordination, while still allowing for personalized interaction when needed.

### 3.3 Pilot Intervention

A total of 39 PCPs completed the pre‐intervention survey, and 36 completed the post‐intervention survey. The mean age of participants was similar pre‐and post‐intervention (39.6 ± 8.1 vs. 39.7 ± 7.9 years), as was years in practice (10.9 ± 7.9 vs. 10.6 ± 7.5 years). The distribution of gender and race remained largely consistent between the two time points.

Significant improvements were observed in comfort identifying patients needing hypercalcemia workup (3.0 ± 1.0 vs. 4.1 ± 0.7, *p* < 0.001), with a 36.7% increase post‐intervention. Familiarity with potential complications of untreated hypercalcemia increased 36.7% from 3.0 ± 1.1 to 4.1 ± 0.7 (*p* < 0.001). Comfort communicating urgency increased 44.4% from 2.7 ± 1.2 to 3.9 ± 0.9 (*p* < 0.001). Referral decisions also improved, as the proportion of PCPs who would refer a standard patient with serum calcium 10.4 mg/dL and PTH 65 pg/mL increased from 34.8% (14/39) pre‐intervention to 78.3% (28/36) post‐intervention (*p* < 0.001). Regarding barriers to referral, lack of familiarity decreased slightly from 41.3% (16/39) to 37.0% (13/36), and perceived low priority decreased from 30.4% (12/39) to 19.6% (7/36). Specialist access as a barrier increased from 42.5% (17/40) to 60.9% (14/23). Other barriers, including unclear guidelines and miscellaneous factors, showed minimal changes. These findings are shown in Table 5.

**Table 5:** PCP survey responses.

Variable	Pre‐intervention (*n* = 39)	Post‐intervention (*n* = 36)	P‐value
Age (Mean ±SD)	39.6 ± 8.1	39.7 ± 7.9	
**Gender**			
Female	28 (60.9%)	28 (60.9%)	
Male	16 (34.8%)	16 (34.8%)	
**Race**			
White	28 (60.9%)	25 (54.3%)	
Black or African American	5 (10.9%)	6 (13.0%)	
Asian or Pacific Islander	7 (15.2%)	8 (17.4%)	
Multiple Races	3 (6.5%)	3 (6.5%)	
Other	3 (6.5%)	4 (8.7%)	
Years in Practice	10.9 ± 7.9	10.6 ± 7.5	
Comfort in identifying patients needing hypercalcemia referral	3.0 ± 1.0	4.1 ± 0.7	< 0.001*
**Would refer patient with Ca = 10.4 and PTH = 65?**			< 0.001*
Yes	16 (34.8%)	36 (78.3%)	
No	11 (23.9%)	4 (8.7%)	
Unsure	19 (41.3%)	6 (13.0%)	
Familiarity with complications of untreated hypercalcemia	3.0 ± 1.1	4.1 ± 0.7	< 0.001*
Comfort communicating urgency of hypercalcemia	2.7 ± 1.2	3.9 ± 0.9	< 0.001*
**Barriers to referral**			
Specialist Access	20 (42.5%)	28 (60.9%)	
Lack of Familiarity	19 (41.3%)	17 (37.0%)	
Unclear Guidelines	18 (39.1%)	14 (30.4%)	
Perceived Low Priority	14 (30.4%)	9 (19.6%)	
Other	0 (0%)	1 (2.2%)	

*p < 0.05 for statistically significant results.

## 4 Discussion

This mixed‐methods study explored the complex, multi‐level contributors to delays in endocrine surgical care and identified opportunities for intervention.

Median referral delay was 157 days compared to median treatment delay of 62 days, suggesting that delays occur predominantly prior to surgical evaluation rather than within surgical specialty care. This distinction is critical as it identifies the referral phase of care as a key barrier to timely care. In endocrine surgery—a highly specialized field where referrals frequently originate outside the treating institution—timely care requires coordination across multiple providers and systems. One study cites patient portals as a potential solution, creating a “loop closure” effect as patients take increased initiative in their care [13]. While portal enrollment was associated with shorter referral delays in univariate analysis, this effect was not sustained after multivariate adjustment, indicating that access alone does not overcome structural and patient‐level barriers.

Instead, our quantitative findings demonstrated that increasing age was associated with both longer referral and treatment delays, which may reflect a combination of factors including greater medical complexity, competing comorbidities, and a higher likelihood of initial nonoperative management or watchful waiting in older patients [14]. Female sex was associated with longer referral delays, which may reflect differences in healthcare‐seeking behavior, symptom communication, or provider‐level bias in escalation of diagnostic workup for thyroid‐related symptoms, although this finding may also be confounded by the higher baseline prevalence of thyroid disease in women and more frequent incidental or indolent presentations [15]. Black or African race was associated with longer treatment delays, consistent with prior literature demonstrating disparities in access to specialty surgical care, including differences in insurance status, referral pathways, geographic proximity to high‐volume endocrine centers, and systemic barriers within healthcare delivery networks [16]. Finally, thyroid disease itself was associated with longer referral and treatment delays, which may reflect the generally indolent nature of many thyroid conditions, leading to initial observation strategies, delayed escalation to surgical consultation, and patient or provider preference for surveillance in the absence of overt compressive symptoms or high‐risk features [17]. Although our quantitative analysis found longer referral and treatment delays among patients with thyroid disease, our qualitative data provided additional context. One staff member noted an informal practice of expediting suspected thyroid cancer referrals, with patients typically scheduled to be seen within approximately 3 weeks.

Qualitative findings provide further nuanced insights for these delay results. Patients described difficulty navigating the referral process, including uncertainty regarding next steps, limited access to direct communication with clinical staff, and lack of anticipatory guidance. These barriers were compounded by the emotional burden of a new diagnosis. Providers echoed this sentiment, citing unclear referral pathways, fragmented communication channels, and lack of standardized protocols as challenges [18]. Together, these findings suggest that delays are not attributable to a single failure point but rather arise from cumulative inefficiencies across patient‐, provider‐, and system‐level domains.

Our findings are particularly salient in delays for patients with primary hyperparathyroidism. Prior literature demonstrates that up to 50% of elevated calcium levels are missed by primary care providers, as they are underdocumented or attributed to other causes [19]. Even when calcium and parathyroid hormone (PTH) are abnormal, 40% of diagnoses are delayed or missed, especially when patients present with minimal or generalizable symptoms [20]. Only 20% are referred for surgery, and those who are referred wait a median of 16 months before seeing a surgeon. Parathyroidectomy is curative, yet untreated PHPT can lead to osteoporosis, nephrolithiasis, renal dysfunction, and neurocognitive symptoms. Our interview participants lived through these statistics firsthand, as one patient expressed frustration that it took 20 years since their first hypercalcemia value for a physician to diagnose them with PHPT. Our interview findings suggested that delays in PHPT are more often driven by missed recognition of hypercalcemia due to human errors in diagnosis rather than disease indolence as in the case of thyroid diseases, which informed the selection of PHPT as the topic for our intervention.

To address this gap, we implemented a pilot intervention to bridge the gap between primary care providers and endocrine surgeons. Our educational intervention resulted in significant improvements in recognition of hypercalcemia, understanding of disease complications, and likelihood of accurate referral. These findings are consistent with prior work demonstrating that referring physicians who communicated directly with specialists had threefold higher odds of referral completion [21].

Yet, education alone is unlikely to fully address these delays. Emerging strategies such as electronic consultation (eConsult) systems offer additional opportunities to streamline referral pathways by triaging surgical candidates more efficiently [22]. Another solution is the development of automated, EHR‐integrated dashboards to identify and track patients with untreated hypercalcemia. The dashboard will alert primary care providers to reach out and schedule an appointment to ensure that diagnoses are not being missed and at‐risk patients are being followed in a timely manner. Advances in artificial intelligence may further support these efforts [23]. However, our qualitative data highlight a critical challenge. Clinicians are already burdened by multiple, fragmented communication platforms. Patient portals and electronic health records (EHR) messaging, while increasingly utilized during the Covid‐19 pandemic, has introduced an additional layer of care delivery outside of traditional clinical encounters, contributing to increased clinician workload and reduced productivity without corresponding reimbursement [24]. Without careful integration into existing workflows, additional tools such as eConsult risk exacerbating cognitive load and staff burnout. Future innovations should therefore focus not only on increasing communication, but on optimizing it. Technology must complement, and not replace human‐centered communication, as patients consistently value direct human interaction in their care.

### 4.1 Limitations

This study has several limitations. Manual chart reviews may contain human errors. Missing or incomplete records also pose a threat to our validity, since data—especially from outside of the MedStar Health System—were difficult to obtain despite databases such as CRISP and CommonWell. Unmeasured factors such as insurance status, education level, health literacy, and comorbidities may have influenced delays. Additionally, portal enrollment was used as a binary proxy for access to technology, which fails to visualize the degree of portal use, including how many messages were sent, how fast the response rates were, and whether the patients were using the portal before referral, during scheduling, post‐operative follow‐up, or not at all. Moreover, the dataset did not allow for differentiation between thyroid cancer and benign thyroid disease, which may have influenced observed delays patterns. Lastly, in our pilot intervention study, the pre‐ and post‐intervention responses could not be matched to protect the identity of the participants, reducing the precision of effect estimates.

## 5 Conclusions

Ultimately, mitigating delays in endocrine surgical care will require coordinated, multi‐level interventions that address both structural inefficiencies and communication gaps. In this mixed‐methods study, we identified substantial delays, particularly in the referral process. While portal enrollment showed an association with shorter referral delays, access to digital tools alone is insufficient to meaningfully reduce delays. Our pilot educational intervention demonstrated that provider‐focused strategies can improve recognition and referral for primary hyperparathyroidism, highlighting the importance of strengthening collaboration between primary care providers and surgical specialists. Future studies are needed to evaluate how patient portals are used in practice and whether they contribute meaningfully to improved care coordination. Prospective studies, including interrupted time series analysis, will be essential to determine whether these interventions lead to sustained, measurable reductions in delays and improvements in patient outcomes. Future work should also focus on expanding and evaluating educational interventions across additional endocrine conditions, including thyroid and adrenal disease, while investigating whether improved referral processes and targeted provider education can shorten referral and treatment delays.

## Synopsis

This mixed‐methods study highlights delays in the referral workflow as a barrier to timely endocrine surgical care, driven by fragmented communication patterns and unclear referral pathways. A provider‐focused educational intervention showed promise in improving recognition and referral for patients with primary hyperparathyroidism, emphasizing the importance of strengthening collaboration between primary care providers and surgical specialists. Ultimately, mitigating delays in endocrine surgical care will require coordinated, multi‐level interventions that address both structural inefficiencies and communication gaps.

## Data Availability

The data that support the findings of this study are available on request from the corresponding author. The data are not publicly available due to privacy or ethical restrictions.
